# A Critical Role of Peptidylprolyl Isomerase A Pseudogene 22/microRNA-197-3p/Peptidylprolyl Isomerase A Axis in Hepatocellular Carcinoma

**DOI:** 10.3389/fgene.2021.604461

**Published:** 2021-03-15

**Authors:** Yuwei Gu, Chao Wang, Shengsen Chen, Jia Tang, Xiaoxiao Guo, Wei Hu, An Cui, Dian Zhang, Kangkang Yu, Mingquan Chen

**Affiliations:** ^1^Department of Infectious Diseases, Huashan Hospital, Shanghai, China; ^2^Emergency Department, Huashan Hospital, Shanghai, China; ^3^Department of Endoscopy, Cancer Hospital of the University of Chinese Academy of Sciences (Zhejiang Cancer Hospital), Hangzhou, China; ^4^Institute of Cancer and Basic Medicine (IBMC), Chinese Academy of Sciences, Hangzhou, China; ^5^Shanghai Medical College of Fudan University, Shanghai, China

**Keywords:** hepatocellular carcinoma, pseudogene, PPIA, chemokine, macrophage

## Abstract

The burden of hepatocellular carcinoma (HCC) worldwide is increasing over time, while the underlying molecular mechanism of HCC development is still under exploration. Pseudogenes are classified as a special type of long non-coding RNAs (lncRNAs), and they played a vital role in regulating tumor-associated gene expression. Here, we report that a pseudogene peptidylprolyl isomerase A pseudogene 22 (PPIAP22) and its parental gene peptidylprolyl isomerase A (PPIA) were upregulated in HCC and were associated with the clinical outcomes of HCC. Further investigation revealed that PPIAP22 might upregulate the expression of PPIA through sponging microRNA (miR)-197-3p, behaving as competing endogenous RNA (ceRNA). PPIA could participate in the development of HCC by regulating mRNA metabolic process and tumor immunity based on the functional enrichment analysis. We also found a strong correlation between the expression levels of PPIA and the immune cell infiltration or the expression of chemokines, especially macrophage, C-C motif chemokine ligand 15 (CCL15), and C-X-C motif chemokine ligand 12 (CXCL12). Our findings demonstrate that the PPIAP22/miR-197-3p/PPIA axis plays a vital role in the progression of HCC by increasing the malignancy of tumor cells and regulating the immune cell infiltration, especially macrophage, through CCL15-CCR1 or CXCL12-CXCR4/CXCR7 pathways.

## Introduction

Hepatocellular carcinoma (HCC, LIHC) is the most common type of primary liver cancer, ranking sixth for incidence and fourth for mortality (Bray et al., 2018). In contrast to many other diseases, the overall burden of HCC worldwide is increasing over time. Due to the frequent recurrence, intrahepatic metastasis of HCC, and the shortage of effective treatments, the prognosis of HCC is not optimistic (Wang et al., 2019; Yang et al., 2019). Meanwhile, the underlying molecular mechanism of HCC development is still under exploration. More and more evidence suggests that the dysregulation of non-coding RNAs (ncRNAs) plays an essential role in HCC during the past few decades. By affecting the signal transduction pathways in cancer cells and the interaction between cancer cells and the microenvironment, ncRNAs mediate HCC development and progression (Pea et al., 2020).

The ncRNAs can be divided into short (short ncRNAs, 19–31 nucleotides), medium (mid-size ncRNAs, 20–200 nucleotides) and long (long ncRNAs, >200 nucleotides) according to length, such as microRNAs (miRNAs, 19-24 bp), snoRNAs (60-300 bp), transcribed ultraconserved regions (T-UCRs, >200 bp), respectively (Esteller, 2011; Anastasiadou et al., 2018). Among them, the molecular mechanisms of long non-coding RNAs (lncRNAs) in tumorigenesis are diverse and essential, including acting as competing endogenous RNA (ceRNA; Thomson and Dinger, 2016). Besides, lncRNAs can also regulate the epigenetics (Aguilo et al., 2011), transcriptomics (Saayman et al., 2016; Gu et al., 2019; Miao et al., 2019), miRNA processing (Wang et al., 2014; Tian et al., 2019), and the stability of mRNA and proteins (Cheadle et al., 2005; Zhang et al., 2020a).

Pseudogenes, a particular type of lncRNAs, have not gained enough attention when compared to others. However, with the tremendous development of in-depth research, multiple pseudogenes have been reported to affect various cell functions in diverse types of human cancers (Xiao-Jie et al., 2015; Lou et al., 2020). Pseudogenes can also regulate gene expression like other types of lncRNAs, playing a vital role in tumorigenesis (Hu et al., 2018).

In this research, we first identified the differentially expressed genes of HCC by analyzing the Cancer Genome Atlas (TCGA) databases. Then, we found a pseudogene and explored its prognostic values in cancer patients, especially in HCC patients. Moreover, we studied its potential functions and molecular mechanisms, thereby establishing a pseudogene–miRNA–mRNA network.

## Materials and Methods

### Human Tissue Samples

Fifty paired tissue samples of HCC patients were collected from Huashan Hospital (Shanghai, China) between 2013 and 2017. The study was approved by ethics committee of Huashan Hospital. The informed consent and agreement were obtained from the patients. These samples were used to analyze the mRNA expression of PPIAP22 and PPIA, and evaluate the correlation with clinicopathologic features.

### Expression Level Analysis

The differentially expressed genes of HCC were obtained by using the “Differential gene analysis” module in Gene Expression Profiling Interactive Analysis (GEPIA). The mRNA expression in different types of cancers and in different stages of HCC patients was obtained using GEPIA by analyzing TCGA and GTEx databases (Tang et al., 2017). The result of immunohistochemistry was obtained from the Human Protein Atlas to verify PPIA protein expression level in HCC tissues and normal liver tissues (Uhlén et al., 2015; Uhlen et al., 2017).

### RNA Extraction and Quantitative Real-Time PCR Assay

Total RNA was extracted from human tissue samples with TRIzol Regent (Life technologies). The quality of RNA was examined by NanoDrop 2000 (Thermo Scientific). Reverse transcription was performed using a FastQuant RT Kit (with gDNase, TIANGEN) or miRcute Plus miRNA First-Strand cDNA Kit (TIANGEN). Then, target mRNA levels were examined by using a SuperReal PreMix Plus Kit (SYBR Green, TIANGEN) or miRcute Plus miRNA qPCR Detection Kit (TIANGEN) and Roche LightCycler480. ACTB was used as an internal control of RNA integrity. Primer sequences:

PPIAP22-F: 5-CAGGGTTTATGTGTCAGGGTGGTG-3.
PPIAP22-R: 5-TGGTGATCTTCTTGCTGGTCTTGC-3.
PPIA-F: 5-TGCTGGACCCAACACAAATG-3.
PPIA-R: 5-AACACCACATGCTTGCCATC-3.
miR-197-3p-F: 5-GTTCACCACCTTCTCCAC-3.

### Survival Analysis

Gene Expression Profiling Interactive Analysis was used to analyze the relationship between PPIAP22 or PPIA gene expression levels and disease-free survival (DFS) or overall survival (OS) of cancer patients. Quartile group cutoff value, 95% CI, log-rank test, and the Cox proportional hazard ratio were used for survival analysis in GEPIA. TIMER was used to analyze the relationship between different immune cell infiltration and OS of HCC patients, in which the tumor purity has been corrected. About 50% group cutoff value, 95% CI, log-rank test, and the Cox proportional hazard ratio were used for survival analysis in TMIER (Li et al., 2017).

### Similarity and Correlation Between PPIAP22 and PPIA

The similarity between PPIAP22 and PPIA was analyzed using the “BLAST” module in NCBI. Moreover, the correlation between PPIAP22 and PPIA was analyzed using the “Correlation analysis” module in GEPIA.

### PPIAP22 Cellular Localization Prediction and Candidate miRNAs Analysis

The sequence of PPIAP22 was obtained from NCBI. LncLocator was used to analyze the cell location of PPIAP22 by its sequence based on a stacked ensemble classifier (Cao et al., 2018). Potential binding miRNAs to PPIAP22 and PPIA were predicted using dreamBase (Zheng et al., 2018), starBase v2.0 (Li et al., 2014), and miRTarBase (Huang et al., 2020). Then the predicted miRNAs were analyzed by Venn diagram. Prediction of target site between PPIAP22 or PPIA and miR-197-3p were analyzed by dreamBase and miRanda (John et al., 2004).

### Correlation Analysis of Gene Expression

The differential genes, co-expressed with PPIA, were predicted by the “LinkFinder” module in LinkedOmics. In this module, the LIHC cohort in TCGA database was analyzed. Pearson’s correlation coefficient was used to identify the co-expressed genes in the LIHC dataset (Vasaikar et al., 2018). The physical interactors of PPIA were identified from BioGRID, containing 1,623,645 proteins and genetic interactions in several species (Oughtred et al., 2019).

### Transfection

About 2 ug plasmid or 50 pmol miR-197-3p inhibitor or mimic and their negative control (NC; GenePharma) was transfected *via* Lipofectamine 3,000 Reagent (Thermo Fisher Scientific) in 12-well plate. The cells were collected for RNA extraction 24 h later.

### Functional Enrichment Analysis

The interactors of PPIA were obtained from BioGRID. Metascape was used to perform the functional enrichment analysis of these genes. For each given gene list in Metascape, protein-protein interaction enrichment analysis has been carried out with the following databases: STRING, BioGRID, OmniPath, and InWeb_IM. Only physical interactions in STRING (physical score > 0.132) and BioGRID are used. The resultant network contains the subset of proteins that form physical interactions with at least one other member in the list. If the network contains between 3 and 500 proteins, the Molecular Complex Detection (MCODE) algorithm has been applied to identify densely connected network components (Zhou et al., 2019).

### Construction of PPIA Interacted Gene Network

The interactors of PPIA were obtained from BioGRID. PPIA interacted gene network was analyzed, established, and visualized with Cytoscape in Metascape. Integrated Value of Influence (IVI) was used to identify the most influential network nodes and visualize the network according to IVI values (Salavaty et al., 2020).

### Correlation Analysis Between PPIA Expression and Immune Cell Infiltration or Chemokines

The correlation between PPIA expression and immune cell infiltration in HCC was visualized using TIMER, in which tumor purity has been corrected. The correlation between PPIA expression and chemokines in HCC were analyzed by TISIDB, an integrated repository portal for tumor immune system interactions (Ru et al., 2019).

### Statistical Analysis

In the bioinformatics analysis, the statistical methods and thresholds used website default parameters. Besides, the experimental data are presented as the mean ± SD. PPIAP22, PPIA, and miR-197-3p expression differences were analyzed by Student’s *t*-test (paired, two-tailed). The 50 pairs of HCC tumor tissues and normal tissues are divided into two groups (low expression and high expression) according to PPIAP22 or PPIA mRNA expression. The correlation between clinicopathologic features and PPIAP22 or PPIA mRNA expression was analyzed by chi-square test or Fisher’s exact test. *p*-values are denoted as follows: ^*^p < 0.05, ^**^p < 0.01, ^***^p < 0.001, and ^****^p < 0.0001. “*p* < 0.05” was defined as statistically significant. Meanwhile, “*p* < 0.01” or “^**^” means *p*-value is between 0.001 and 0.01. Similarly, “*p* < 0.001” or “^***^” means *p*-value is between 0.0001 and 0.001. “*p* < 0.001” or “^****^” means *p*-value is less than 0.0001.

## Results

### Upregulated PPIAP22 and PPIA Predict Poor Prognosis of HCC Patients

**Figure 1:**
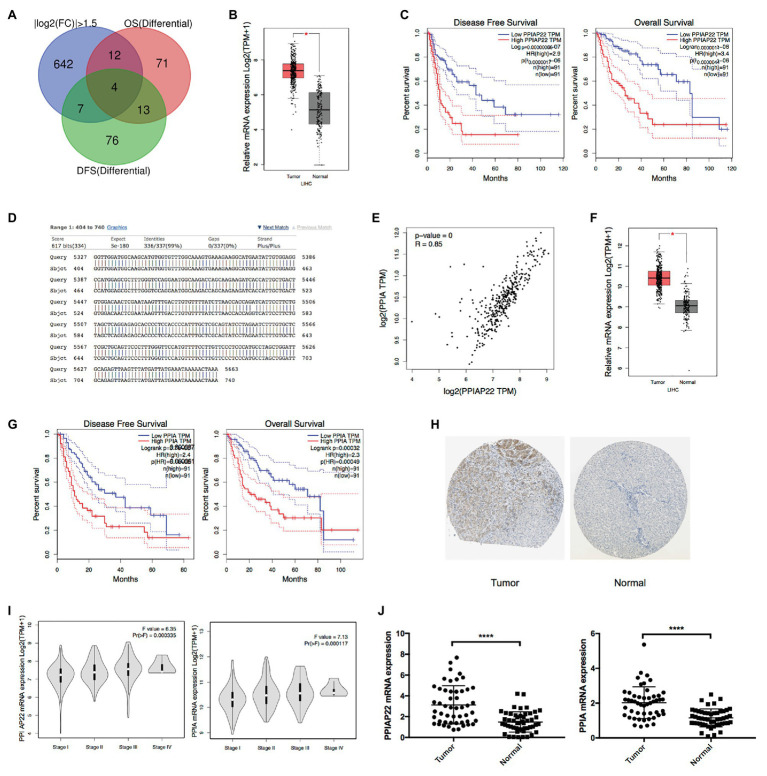
Upregulated peptidylprolyl isomerase A pseudogene 22 (PPIAP22) and peptidylprolyl isomerase A (PPIA) are significantly predicted poor prognosis of hepatocellular carcinoma (HCC) patients. **(A)** Bioinformatics analysis of differential genes with a log2 fold change ≥|1.5|, *p* < 0.05, and with significant associations with overall survival (OS) and disease-fee survival (DFS) of HCC patients using Gene Expression Profiling Interactive Analysis (GEPIA). **(B)** PPIAP22 was upregulated in tumor tissues with HCC compared with normal tissues, analyzed with GEPIA using the Cancer Genome Atlas (TCGA) and GTEx databases. **(C)** Prognostic values of PPIAP22 in HCC analyzed with GEPIA. The dotted lines represent the 95% CI. **(D)** Sequence similarity between PPIAP22 and its parental gene PPIA. **(E)** Correlation analysis between PPIAP22 and PPIA in HCC using GEPIA. **(F)** PPIA was upregulated in tumor tissues with HCC compared with normal tissues, analyzed with GEPIA. **(G)** Prognostic values of PPIA in HCC analyzed with GEPIA using TCGA and GTEx databases. The dotted lines represent the 95% CI. **(H)** The expression of PPIA was analyzed using immunohistochemistry from the Human Protein Atlas database. **(I)** The correlation between PPIAP22 or PPIA mRNA expression and the clinic stage of HCC patients were analyzed with GEPIA. **(J)** PPIAP22 and PPIA mRNA expression levels were higher in tumor tissues than in normal (50 pairs, *p* < 0.0001). PPIAP22, peptidylprolyl isomerase A pseudogene 22; PPIA, peptidylprolyl isomerase A; and HCC, hepatocellular carcinoma. ^*^p < 0.05; ^****^p < 0.0001, respectively.

To investigate the differential genes in HCC, the TCGA database was examined by GEPIA database analysis. Four genes, KPNA2, PPIAP22, HN1, ZWINT, were identified with log2 fold change ≥|1.5|, *p* < 0.05, and with significant associations with the OS and DFS of HCC patients (Figure 1A). Among the genes, PPIAP22, a pseudogene, was significantly upregulated in tumor tissues of HCC compared with normal tissues (Figure 1B). Survival analysis using Kaplan-Meier plotter suggested that high expression levels of PPIAP22 in patients with HCC exhibited significantly more reduced DFS (*p* < 0.001; Figure 1C) and more reduced OS (*p* < 0.001; Figure 1C). To further investigate the role of PPIAP22 in the progression of HCC, its sequence in the human genome was first blasted using NCBI. We found a 99% similarity, based on their sequence, between PPIAP22 and its parental gene peptidylprolyl isomerase A (also called cyclophilin A, PPIA; Figure 1D). A strong positive relationship was revealed between PPIAP22 and PPIA in HCC visualized by Pearson correlation analysis (Figure 1E). Using GEPIA database, we observed an increased mRNA expression of PPIA in tumor tissues of HCC compared with normal tissues (Figure 1F). PPIA in patients with HCC also exhibited significantly more reduced DFS (*p* < 0.001; Figure 1G) and more reduced OS (*p* < 0.001; Figure 1G). The results above suggest that PPIAP22 in HCC may play a vital role in regulating the expression of its parental gene PPIA.

We also observed a much higher level of PPIA expression in tumor tissues than in normal using immunohistochemistry from the Human Protein Atlas database (Figure 1H). Besides, both the level of PPIA expression and PPIAP22 expression were also positively correlated with the clinical stage of HCC patients (*p* < 0.001; Figure 1I).

To further confirm the mRNA level of PPIAP22 and PPIA, we analyzed 50 pairs tissue samples of HCC patients from our hospital. PPIAP22 and PPIA mRNA expression levels were also significantly higher in tumor tissues than in normal tissues (*p* < 0.0001; Figure 1J). We also analyzed the correlation between expression of PPIAP22 or PPIA and clinicopathologic features, including gender, age, tumor size, number of tumors, metastasis, and TMN stages. Only number of tumors exhibited significantly correlation with PPIAP22 or PPIA mRNA expression level (*p* < 0.05; Supplementary Figures S1, S2).

### PPIAP22 and PPIA Play Significant Roles in Other Types of Cancer

**Figure 2:**
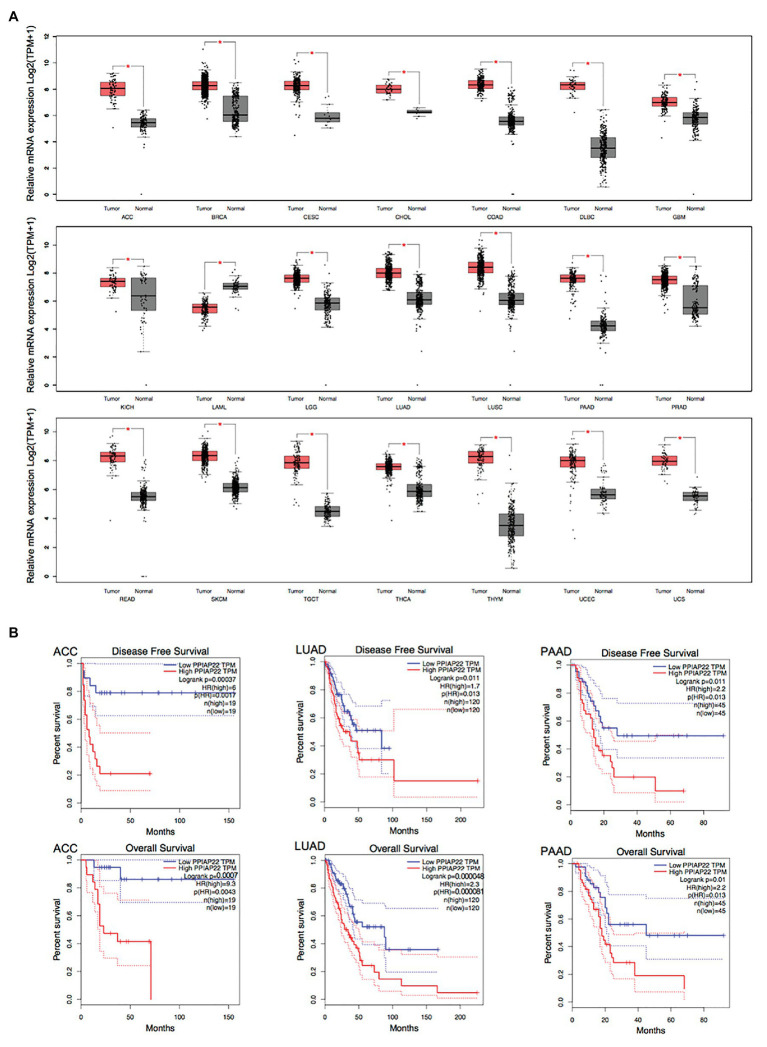
Peptidylprolyl isomerase A pseudogene 22 plays a significant role in other types of cancer. **(A)** PPIAP22 was dysregulated in other types of cancer identified by using TCGA and GTEx databases through GEPIA. **(B)** Prognostic values of PPIAP22 in adrenocortical carcinoma (ACC), lung adenocarcinoma (LUAD), and pancreatic adenocarcinoma (PAAD) analyzed with GEPIA. The dotted lines represent the 95% CI. PPIAP22, peptidylprolyl isomerase A pseudogene 22; ACC, adrenocortical carcinoma; LUAD, lung adenocarcinoma; and PAAD, pancreatic adenocarcinoma. ^*^p < 0.05.

**Figure 3:**
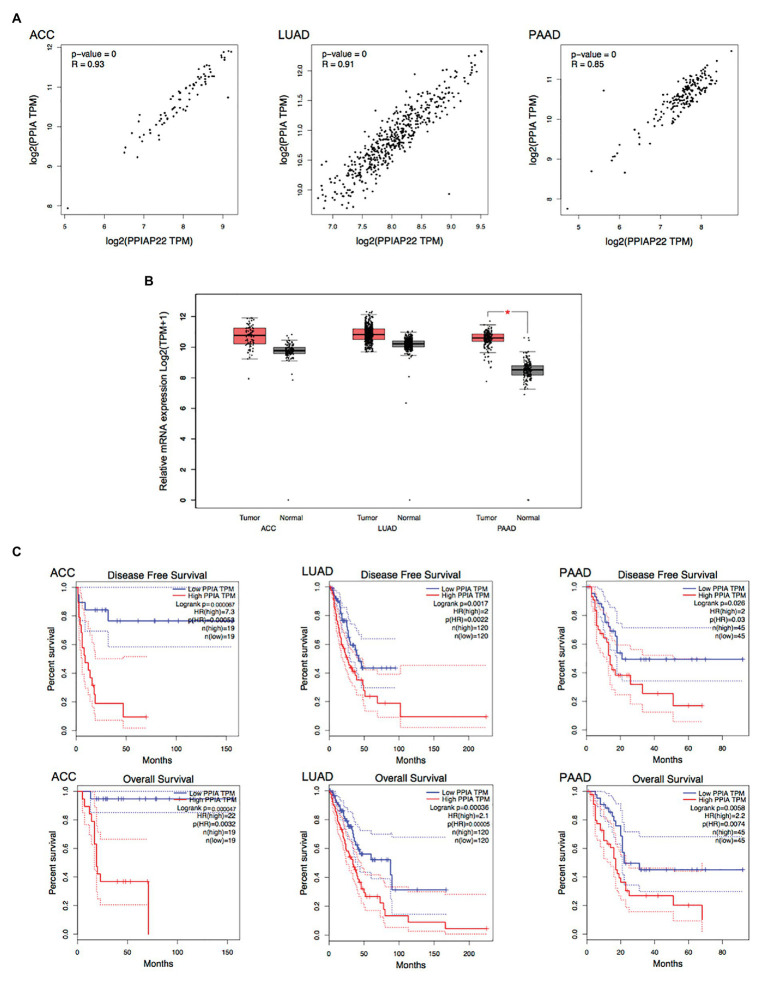
Peptidylprolyl isomerase A plays a significant role in other types of cancer. **(A)** Correlation analysis between PPIAP22 and PPIA in ACC, LUAD, and PAAD using GEPIA. **(B)** PPIA was upregulated in ACC, LUAD, and PAAD identified by using TCGA and GTEx databases through GEPIA. **(C)** Prognostic values of PPIA in ACC, LUAD, and PAAD analyzed with GEPIA. The dotted lines represent the 95% CI. PPIAP22, peptidylprolyl isomerase A pseudogene 22; PPIA, peptidylprolyl isomerase A; ACC, adrenocortical carcinoma; LUAD, lung adenocarcinoma; and PAAD, pancreatic adenocarcinoma. ^*^p < 0.05.

We found that PPIAP22 was also dysregulated in other types of cancer. The PPIAP22 expression was increased in adrenocortical carcinoma (ACC), breast invasive carcinoma (BRCA), cervical squamous cell carcinoma and endocervical adenocarcinoma (CESC), cholangiocarcinoma (CHOL), colon adenocarcinoma (COAD), lymphoid neoplasm diffuse Large B-cell Lymphoma (DLBC), glioblastoma multiforme (GBM), kidney Chromophobe (KICH), brain lower grade glioma (LGG), lung adenocarcinoma (LUAD), lung squamous cell carcinoma (LUSC), pancreatic adenocarcinoma (PAAD), prostate adenocarcinoma (PRAD), rectum adenocarcinoma (READ), skin cutaneous melanoma (SKCM), testicular germ cell tumors (TGCT), thyroid carcinoma (THCA), thymoma (THYM), uterine corpus endometrial carcinoma (UCEC), and uterine carcinosarcoma (UCS), while the mRNA expression of which was decreased in acute myeloid leukemia (LAML; *p* < 0.05; Figure 2A). High expression levels of PPIAP22 were related to worse DFS and OS of patients with ACC, LUAD, and PAAD (Figure 2B). A strong positive relationship between PPIAP22 and PPIA in ACC, LUAD, and PAAD was revealed by Pearson correlation analysis (Figure 3A). Besides, the mRNA expression of PPIA was also upregulated in ACC, LUAD, and PAAD, among which only PAAD has significant differences (Figure 3B). The high-level expression of PPIA was also related to worse overall survival of patients with ACC, LUAD, and PAAD (Figure 3C). These data indicate that PPIAP22 may play a vital role in regulating PPIA expression in PAAD apart from HCC.

### PPIAP22 May Function as ceRNA Competing With PPIA for miR-197-3p Binding

**Figure 4:**
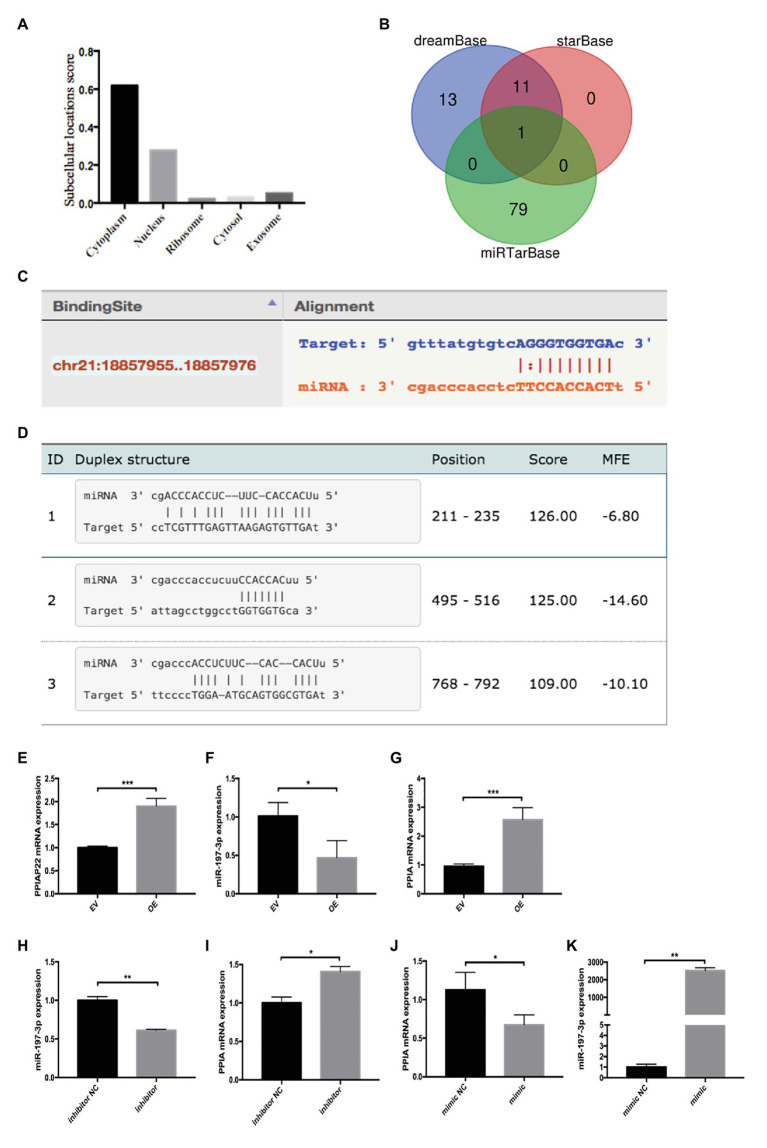
Peptidylprolyl isomerase A pseudogene 22 may function as competing endogenous RNA (ceRNA) competing with PPIA for miR-197-3p binding. **(A)** Cell localization of PPIAP22 predictied by lncLocator. **(B)** Bioinformatics analysis of PPIAP22 and PPIA candidate microRNAs (miRNAs). **(C)** Binding sites between miR-197-3p and PPIAP22 predicted by dreamBase. **(D)** Binding sites between miR-197-3p and PPIA predicted by miRanda. **(E)** Overexpression of PPIAP22 in MHCC-97H was detected by RT-qPCR. **(F)** Overexpression of PPIAP22 in MHCC-97H resulted in decreased expression of miR-197-3p. **(G)** Overexpression of PPIAP22 in MHCC-97H resulted in increased expression of PPIA. **(H)** The expression of miR-197-3p in MHCC-97H transfected with negative control (NC) or miR-197-3p inhibitor. **(I)** The expression of PPIA was increased in the miR-197-3p inhibitor group. **(J)** The expression of PPIA was decreased in the miR-197-3p mimic group. **(K)** The expression of miR-197-3p in MHCC-97H transfected with negative control or miR-197-3p mimic. PPIAP22, peptidylprolyl isomerase A pseudogene 22; PPIA, peptidylprolyl isomerase A; miR, microRNA; ceRNA, competing endogenous RNA; EV, empty vector; and NC, negative control. ^*^p < 0.05; ^**^p < 0.01; ^***^p < 0.001, respectively.

The cell localization of lncRNAs provides essential information for understanding their complex biological functions. Apart from the cytoplasm, PPIAP22 also locates in the nucleus and exosome (Figure 4A), indicating that it may regulate PPIA expression *via* the ceRNA mechanism. DreamBase, starBase, and miRTarBase predicated miR-197-3p as candidate miRNA of PPIAP22 and PPIA (Figure 4B). The binding sites were predicted by dreamBase (Figure 4C) and miRanda (Figure 4D). We further found that the expression of miR-197-3p in HCC tumor tissues was downregulated compared with matched adjacent non-tumor tissues. Meanwhile, the DFS and OS of the low miR-197-3p subgroup were significantly reduced divided from the high miR-197-3p subgroup (Ni et al., 2019). The results above indicated that PPIAP22 might function as a ceRNA to promote the expression of PPIA through binding to miR-197-3p.

Next, we overexpress PPIAP22 by plasmid transfection in MHCC-97H cells (Figure 4E; *p* < 0.001), a HCC cell line. We found that overexpression of PPIAP22 in MHCC-97H cells resulted in decreased expression of miR-197-3p (Figure 4F; *p* < 0.05) and increased expression of PPIA (Figure 4G; *p* < 0.001). We also modulated the expression of miR-197-3p in MHCC-97H cells with NC or miR-197-3p inhibitor (Figure 4H; *p* < 0.01). The expression of PPIA was increased in the miR-197-3p inhibitor group (Figure 4I; *p* < 0.05). And the expression of PPIA was decreased (Figure 4J; *p* < 0.05) when we transfected with miR-197-3p mimic (Figure 4K; *p* < 0.01). Here, we confirmed that PPIAP22 could function as ceRNA to promote the expression of PPIA through binding to miR-197-3p.

### Enrichment Analysis of PPIA Functional Networks in HCC

**Figure 5:**
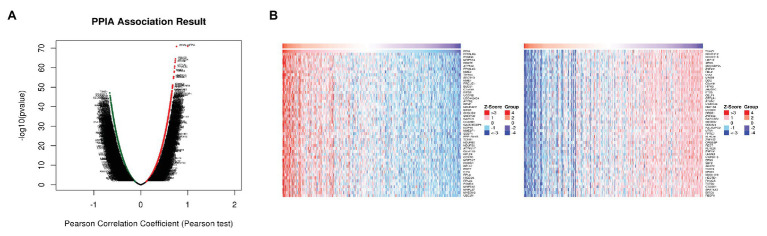
Differentially expressed genes related to PPIA in HCC. **(A)** The correlation between PPIA and the differentially expressed genes in HCC analyzed using Pearson test. The red dots indicate positively related genes, and the green dots indicate negatively related genes. **(B)** Heat maps showing genes (top 50) that are positively correlated (left) and negatively correlated (right) with PPIA in HCC. PPIA, peptidylprolyl isomerase A; HCC, hepatocellular carcinoma.

**Figure 6:**
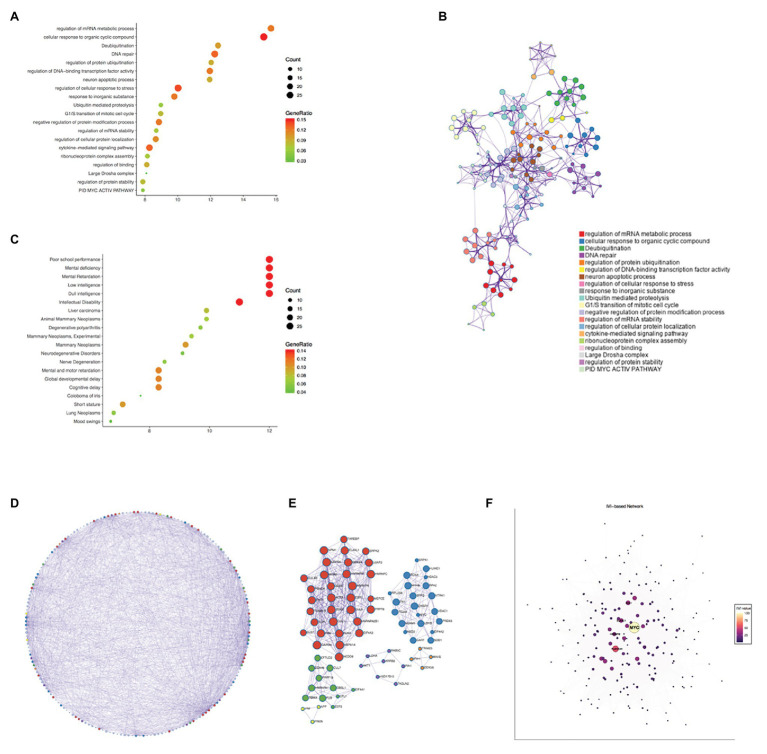
Enrichment analysis of PPIA functional networks in HCC. **(A)** Enrichment analysis of PPIA related genes using Metascape. The nodes represent different enriched terms. The size of nodes represent the count of genes and the color represent the GeneRatio. **(B)** The network of enriched terms colored by cluster ID, the nodes that share the same cluster ID are typically close to each other. The size of nodes represent the count of genes. **(C)** Enrichment analysis of gene-disease association using Metascape. The nodes represent different enriched terms. The size of nodes represent the count of genes and the color represent the GeneRatio. **(D,E)** Protein-protein interaction network carried out with Metascape and gene lists in Molecular Complex Detection (MCODE) networks. The nodes were colored by MCODE, and nodes that share the same MCODE are typically close to each other. The size of nodes represent the count of genes. **(F)** The visualization of network according to Integrated Value of Influence (IVI) values. The nodes represent different co-expressed genes. A centrality measure was applied to the size and color of network nodes according to IVI values. PPIA, peptidylprolyl isomerase A; HCC, hepatocellular carcinoma; and MCODE, the Molecular Complex Detection.

The genes co-expressed with PPIA were identified by LinkedOmics through analyzing mRNA sequencing data from 371 LIHC patients in the TCGA. The 3,950 genes represented by the red dots are significantly positively correlated with PPIA, while the 5,909 genes represented by the green dots are significantly negatively correlated (Figure 5A). The heatmap shows the top 50 significant gene sets that are positively and negatively correlated with PPIA, respectively (Figure 5B), exhibiting the extensive impact of PPIA on the transcriptome. To further explore its function, the interactors of PPIA were determined using BioGRID, leading to the identification of 208 physical interactors with various experimental systems. Metascape enrichment analysis of 194 PPIA interactors belonging to *Homo sapiens* was performed. The top 20 enriched terms were as follows: “regulation of mRNA metabolic process,” “cellular response to organic cyclic compound,” “Deubiquitination,” “DNA repair,” “regulation of protein ubiquitination,” “regulation of DNA-binding transcription factor activity,” “neuron apoptotic process,” “regulation of cellular response to stress,” “response to inorganic substance,” “Ubiquitin mediated proteolysis,” “G1/S transition of mitotic cell cycle,” “negative regulation of protein modification process,” “regulation of mRNA stability,” “regulation of cellular protein localization,” “cytokine-mediated signaling pathway,” “ribonucleoprotein complex assembly,” “regulation of binding,” “Large Drosha complex,” “regulation of protein stability,” and “PID MYC ACTIV PATHWAY” (Figures 6A,B). Gene list enrichments were also identified in DisGeNET. The liver carcinoma ranks seventh in the enrichment analysis of gene-disease association (Figure 6C).

Next, the protein-protein interaction network was carried out with Metascape through analyzing BioGrid, InWeb_IM, and OmniPath databases, and the MCODE algorithm has been used to exhibit closely connected network components (Figure 6D). The genes in different MCODE networks were also listed (Figure 6E).

According to degree analyzed in Metascape, the top 10 interacted genes in the network were listed (Table 1), which were MYC, PLEKHA4, EFTUD2, ACTB, PHB, RECQL4, HSPA1A, ELAVL1, HNRNPA1, and PKM. Besides, we used IVI to identify the most influential network nodes. The top 10 influential network nodes were MYC, PLEKHA4, CUL1, ELAVL1, HNRNPM, TRIM25, ACTB, VIRMA, CFL1, and EEF2 (Table 2). The visualization of network was based on applying a centrality measure to the size and color of network nodes according to IVI values (Figure 6F). MYC showed the highest node degree and IVI value in both methods above, which were 102 and 100, respectively.

**Table 1:** The top 10 interacted genes in the network according to degree analyzed in Metascape.

Rank	Gene symbol	Degree
1	MYC	102
2	PLEKHA4	86
3	EFTUD2	78
4	ACTB	76
5	PHB	74
6	RECQL4	73
7	HSPA1A	72
8	ELAVL1	72
9	HNRNPA1	71
10	PKM	70

**Table 2:** The top 10 influential network nodes according to IVI value analyzed by IVI.

Rank	Gene symbol	IVI value
1	MYC	100
2	PLEKHA4	54
3	CUL1	34
4	ELAVL1	33
5	HNRNPM	33
6	TRIM25	32
7	ACTB	32
8	VIRMA	32
9	CFL1	30
10	EEF2	30

Pathway and process enrichment analysis have been applied to each MCODE component independently. The three best matching items obtained through the *p*-value are retained as the functional description of the corresponding components, which were “regulation of mRNA metabolic process,” “RNA splicing,” “nucleobase-containing compound catabolic process” in the red MCODE, and “leukocyte differentiation,” “PID INTEGRIN5 PATHWAY,” “lymphocyte activation” in the blue one. The top three terms in the green MCODE were “mRNA Splicing – Major Pathway,” “mRNA Splicing” and “Processing of Capped Intron-Containing Pre-mRNA.” In purple were “negative regulation of protein binding,” “negative regulation of binding,” and “positive regulation of apoptotic process,” in orange one were “NF-kB activation through FADD/RIP-1 pathway mediated by caspase-8 and -10,” “TRAF3-dependent IRF activation pathway” and “TRAF6 mediated NF-kB activation,” and in yellow were “cellular response to organonitrogen compound,” “cellular response to nitrogen compound,” and “regulation of neuron differentiation.” Combined with the two respects of enrichment analysis, gene and protein, the regulation of mRNA metabolic process and some pathways involved in tumor immunity had a prominent significance among the function of PPIA.

### The PPIAP22/miR-197-3p/PPIA Correlates With Immune Cell Infiltration and Chemokines in HCC

**Figure 7:**
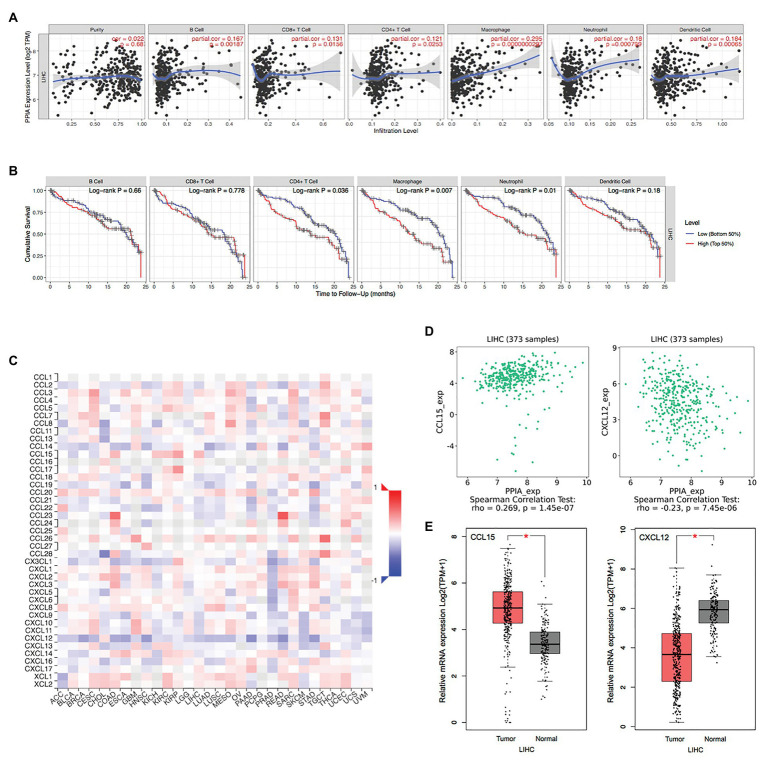
The PPIAP22/miR-197-3p/PPIA is correlated with immune cell infiltration and chemokines in HCC. **(A)** Correlation of PPIA expression with immune cell infiltration in HCC analyzed using TIMER. **(B)** Kaplan-Meier plots for immune cell infiltration and overall survival of HCC were visualized using TIMER. **(C)** Correlation of PPIA expression with chemokines in HCC analyzed using TISIDB. **(D)** Correlation of PPIA expression with C-C motif chemokine ligand 15 (CCL15) and C-X-C motif chemokine ligand 12 (CXCL12) in HCC analyzed using TISIDB. **(E)** CCL15 was upregulated in tumor tissues with HCC and CXCL12 was deregulated compared with normal tissues, analyzed with GEPIA using TCGA and GTEx databases. PPIAP22, peptidylprolyl isomerase A pseudogene 22; PPIA, peptidylprolyl isomerase A; miR, microRNA; HCC, hepatocellular carcinoma; CCL15, C-C motif chemokine ligand 15; and CXCL12, C-X-C motif chemokine ligand 12. ^*^p < 0.05.

To further investigate the function of the PPIAP22/miR-197-3p/PPIA axis in tumor immunity, we analyzed the expression of PPIA and immune cell infiltration in HCC then by using TIMER. A strong correlation was shown in B cell, CD8+ T cell, CD4+ T cell, macrophage, neutrophil, and dendritic cell (Figure 7A), and the immune cell infiltration in HCC is related to the clinical outcome. High levels of CD4+ T cell, macrophage and neutrophil predicted the worst outcome of HCC patients (*p* < 0.05; Figure 7B). These findings indicate that the PPIAP22/miR-197-3p/PPIA axis could affect the clinical outcome of HCC through regulating the tumor immune subsets. The correlations between PPIA expression and chemokines’ expression across HCC were analyzed by TISIDB (Figure 7C). The two most relevant chemokines are C-C motif chemokine ligand 15 (CCL15), showing a significantly positive correlation with PPIA, and C-X-C motif chemokine ligand 12 (CXCL12), showing a significantly negative correlation (*p* < 0.05; Figure 7D). Moreover, the expression level of CCL15 is upregulated in tumor tissues of HCC, and the expression level of CXCL12 is deregulated in HCC (*R* = 0.269, *p* = 1.45e-07; *R* = −0.23, *p* = 7.45e-06, respectively; Figure 7E), which are consistent with PPIA expression changes.

## Discussion

The term “pseudogene” was first used in 1977 when a copy of the 5S rRNA gene was discovered in *Xenopus laevis*. Its 5’end was truncated and 14 bp mismatched, rendering it nonfunctional (Jacq et al., 1977). A large number of pseudogenes were found from then on, considered “junk genes,” “relics of evolution,” or “genomic fossil” (Lou et al., 2020). With subsequent research on pseudogenes, their potential functions were discovered. They play an essential role in various physiological and pathological processes at the DNA, RNA, and protein levels, especially in cancer (Xiao-Jie et al., 2015). Pseudogenes are also crucial components of lncRNAs and can act as key regulators in the occurrence and development of human cancers through sponging miRNAs, functioning as ceRNA (Lou et al., 2020).

In this study, we found four genes in terms of differential expression and impact on clinical outcomes in HCC, including KPNA2, PPIAP22, HN1, and ZWINT. PPIAP22, also known as PPIA3L or PPIAL3, is a pseudogene located in chromosome 21 with a length of 740 bp. We analyzed the mRNA expression level of PPIAP22 in 32 types of cancer, and found that PPIAP22 was upregulated in ACC, BRCA, CESC, CHOL, COAD, DLBC, GBM, KICH, LGG, LIHC, LUAD, LUSC, PAAD, PRAD, READ, SKCM, TGCT, THCA, THYM, UCEC, and UCS, and was deregulated in LAML. Among these cancers, only ACC, LIHD, LUAD, and PAAD showed differential relationships between PPIAP22 expression and the DFS or OS of patients. Thus, PPIAP22 exhibits a widespread impact on cancers and related survival time.

Peptidylprolyl isomerase A, also called cyclophilin A, is the parental gene of PPIAP22 with a similarity of 99%. Our data also suggested that PPIAP22 may function as ceRNA to upregulate PPIA expression through sponging miR-197-3p. Among the cancer types of ACC, LIHD, LUAD, and PAAD, all of them showed high expression levels compared with normal tissues, and only patients of LIHC and PAAD exhibited differential DFS and OS by analyzing the TCGA databases. Besides, we observed a much higher level of PPIA expression in tumor tissues than in normal by using immunohistochemistry. Moreover, there was a positive correlation between the PPIAP22 or PPIA mRNA expression and the clinical stage of HCC patients. However, only number of tumors showed significant correlation with expression of PPIAP22 or PPIA when we analyzed the correlation between expression of PPIAP22 or PPIA and clinicopathologic features, including gender, age, tumor size, number of tumors, metastasis, and TMN stages. We considered that the consequence was somewhat biased because the size of our tissue samples was too small. We still need to collect more tissue samples to clarify the relationship between expression of PPIAP22 or PPIA and clinicopathologic features.

Peptidylprolyl isomerase A as one of the most abundant members of cyclophilin (Cyp) family proteins has been reported upregulated in breast cancer (Volker et al., 2018), esophageal cancer (Qi et al., 2008), small cell lung cancer (Campa et al., 2003), colorectal cancer (Wong et al., 2008), pancreatic cancer (Li et al., 2013), melanoma (Caputo et al., 2011), malignant glioblastoma (Sun et al., 2011; Saw et al., 2018), serous ovarian cancer (Qi et al., 2019), and skin cancer (Han et al., 2012). Cyps possess a peptidyl prolyl isomerase (PPIase), catalyzing the conversion between cis-trans isomers of proline on the peptide (Lee, 2013). PPIase is regarded as a chaperone and can work with other chaperone proteins (Luban, 1996). Besides, PPIA has been found to respond to immune and inflammatory stimuli and considered to be an important protein recently (Nigro et al., 2013; Sánchez et al., 2016).

To further investigate the underlying function of the PPIAP22/miR-197-3p/PPIA axis in cancers, we found 3,950 genes were significantly positively correlated with PPIA, while 5,909 genes were significantly negatively correlated. Our data also showed that liver carcinoma ranks seventh in the enrichment analysis of gene-disease association. Besides, the protein-protein interaction network was carried out with Metascape, and MYC showed the highest node degree. IVI was also used to identify the most influential network nodes. MYC was identified as the most influential network node, which finally exhibited the same results with Metascape. MYC consists of three family members, including MYC, MYCN, and MYCL (Stine et al., 2015; Chen et al., 2018). MYC has been proved to have a board range of functions, including but not limited to cell biology, cell cycle, apoptosis, the molecular basis of cancer (Meyer and Penn, 2008). Meanwhile, MYC has been found involved in the tumorigenesis or progression in neuroblastoma and genotype-C-HBV-related HCC *via* regulating the PPIA expression (Iizuka et al., 2006; Zhang et al., 2020b). In this regard, PPIA may be regulated by MYC to some extent in HCC, apart from PPIAP22.

The enrichment analysis of protein-protein interaction showed a prominent role of PPIA in regulating mRNA metabolic process and some pathways involved in tumor immunity. Several studies have proved that PPIA is related to the regulation of the immune system, and it can have many applications, such as cancer treatment and various processes involving inflammation (Sánchez et al., 2016). In breast cancer, the pharmacological inhibition of PPIA may provide promising and unexpected results in cancer biology, in part by targeting the PPIA/CrkII axis that can modulate the host antitumor immune escape (Davra et al., 2020). However, the actual role of PPIA in HCC and other cancer-related immunity still lacks verification.

Next, we found a strong correlation between PPIA expression and immune cell infiltration in B cells, CD8+ T cells, CD4+ T cells, macrophages, neutrophils, and dendritic cells. Furthermore, infiltration levels of CD4+ T cell, macrophage, and neutrophil are related to a worse outcome of HCC patients. Among the infiltrated immune cells, macrophages exhibited the most remarkable correlation with PPIA expression and prognosis of HCC patients. To investigate the mechanism between the expression level of PPIA and the infiltration level of macrophages in HCC, we analyzed the correlation between PPIA expression and chemokines’ expression. We found that the two most relevant chemokines are CCL15 and CXCL12 in HCC. Besides, the expression level of them is also consistent with PPIA expression changes.

C-C motif chemokine ligand 15 as a member of CC chemokine family binds mainly to CCR1 as a chemoattractant for monocytes, neutrophils, lymphocytes, and dendritic cells (Forssmann et al., 2001) and it is mainly expressed in the intestine and liver (Pardigol et al., 1998). Accumulating evidence suggested that CCL15 plays an essential role in several types of cancer, including lung cancer, colorectal cancer, and hepatocellular carcinoma (Reckamp et al., 2008; Bodelon et al., 2013; Inamoto et al., 2016; Li et al., 2016). CXCL12 is a member of the CXC-chemokine family, mainly binds to CXCR4 and CXCR7, and induces the recruitment of monocyte, myeloid-derived suppressor cells (MDSCs), B cells, and plasmacytoid dendritic cells (pDCs). CXCL12 can affect tumor cells by promoting proliferation, invasion, metastasis, stemness, and modulating the immune response (Scala, 2015; Nagarsheth et al., 2017). The latest data suggest that inhibiting the interaction between CXCR4 expressing T cells and CXCL12 secreting cells in the microenvironment may modulate anti-CTLA-4 or anti-PD-1 immunotherapy in pancreatic cancer and HCC (Feig et al., 2013; Chen et al., 2015). These results demonstrate that the PPIAP22/miR-197-3p/PPIA axis might not only induce the malignancy of tumor cells through CCL15-CCR1 and CXCL12-CXCR4/CXCR7 pathways but also regulate the macrophage infiltration in HCC by recruiting monocytes through CCL15-CCR1 and CXCL12-CXCR4/CXCR7 pathways.

In general, our results suggest a critical role of the PPIAP22/miR-197-3p/PPIA axis in the progression of HCC by increasing malignancy of tumor cells and regulating the immune cell infiltration, especially macrophage, through the CCL15-CCR1 or CXCL12-CXCR4/CXCR7 pathway. The potential roles of the axis in carcinogenesis are worthy of further study. More importantly, the analysis of the pseudogene-miRNA-mRNA ceRNA network may provide potential directions for the treatment of HCC.

## Data Availability Statement

The original contributions presented in the study are included in the article/Supplementary Material, further inquiries can be directed to the corresponding authors.

## Author Contributions

MC and KY designed the study and revised the manuscript. YG and CW performed the TCGA database analysis and wrote the manuscript. SC performed the bioinformatics analysis. JT, XG, WH, AC, and DZ assisted with the collection and analysis of other data. All authors contributed to the article and approved the submitted version.

### Conflict of Interest

The authors declare that the research was conducted in the absence of any commercial or financial relationships that could be construed as a potential conflict of interest.

The reviewers YC and C-YW declared a shared affiliation, with no collaboration, with several of the authors YG, CW, JT, XG, WH, AC, DZ, KY, and MC to the handling editor at the time of the review.

## Supplementary Material

Supplementary figure s1 The correlation between expression of PPIAP22 and clinicopathologic features.

Supplementary Figure S2 The correlation between expression of PPIA and clinicopathologic features.
