# Supplementary figures and images for: A Critical Role of Peptidylprolyl Isomerase A Pseudogene 22/microRNA-197-3p/Peptidylprolyl Isomerase A Axis in Hepatocellular Carcinoma
Source: Front Genet. 2021 Mar 15;12:604461. doi: 10.3389/fgene.2021.604461 (PMC8006304; doi:10.3389/fgene.2021.604461)

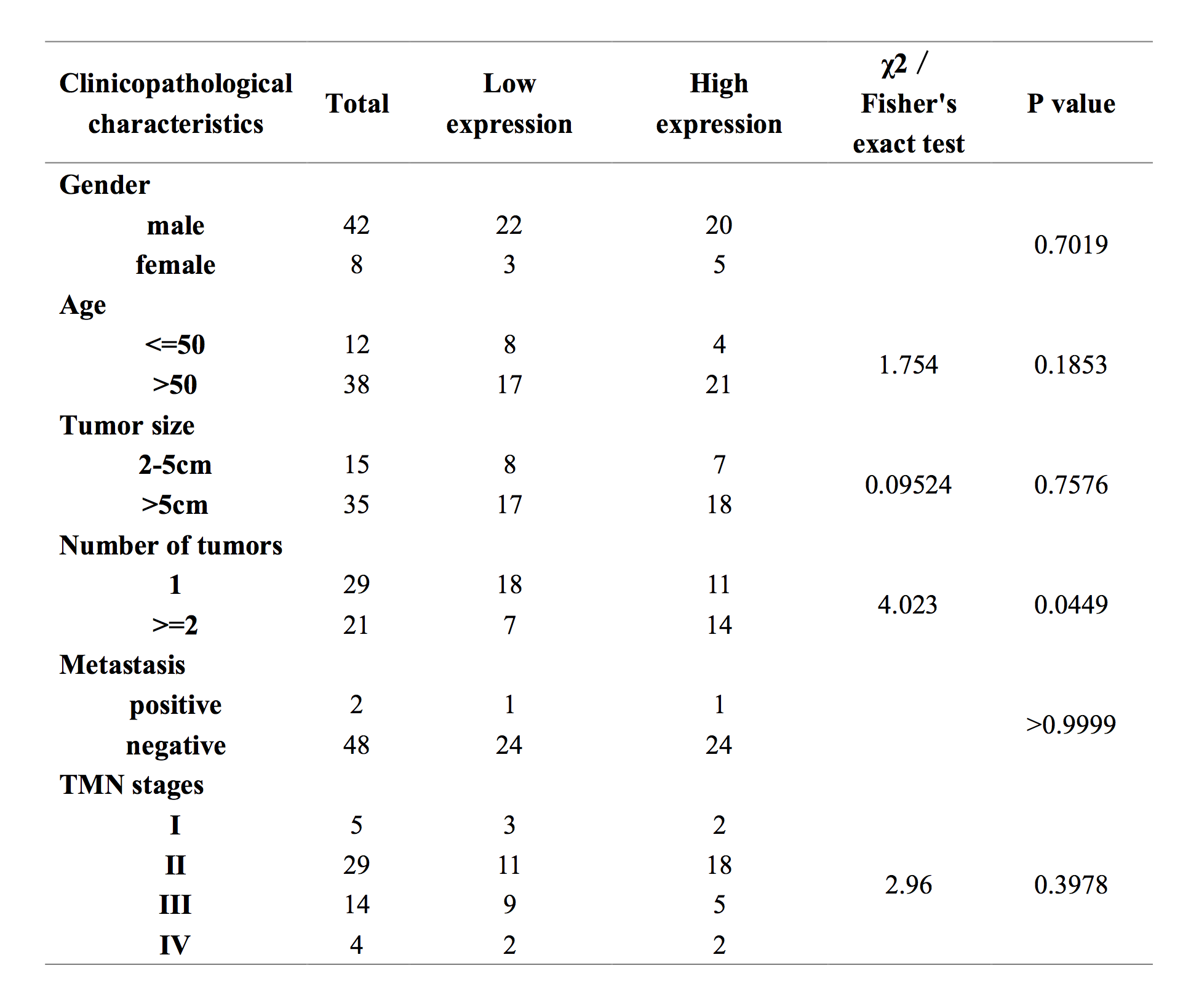

Supplement: Supplementary figure s1 — The correlation between expression of PPIAP22 and clinicopathologic features. [file Image_1.tiff]

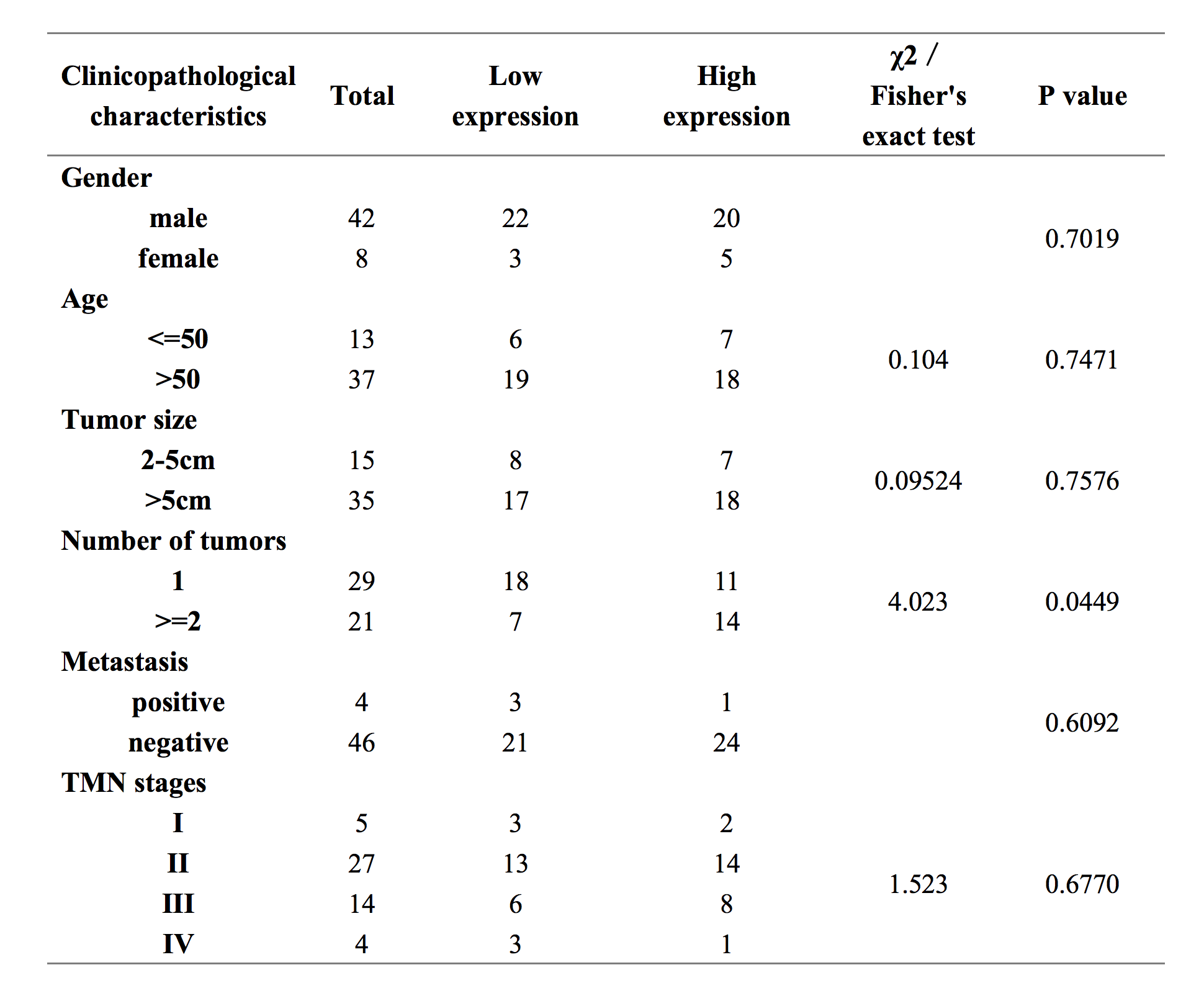

Supplement: Supplementary Figure S2 — The correlation between expression of PPIA and clinicopathologic features. [file Image_2.tiff]
